# Effective Biodegradation of Mycotoxin Patulin by Porcine Pancreatic Lipase

**DOI:** 10.3389/fmicb.2018.00615

**Published:** 2018-04-09

**Authors:** Bingjie Liu, Xiaoning Peng, Xianghong Meng

**Affiliations:** College of Food Science and Engineering, Ocean University of China, Qingdao, China

**Keywords:** porcine pancreatic lipase, patulin, biodegradation, characterization, molecular structure

## Abstract

Patulin is a common contaminant in fruits and vegetables, which is difficult to remove. In this study, the biodegradation of patulin using porcine pancreatic lipase (PPL) was investigated. The method of HPLC was used to analyze the concentration of patulin. Batch degradation experiments were performed to illustrate the effect of PPL amount, pH, temperature, contact time, and initial concentration. Besides, the degradation product of patulin was characterized by full wavelength scanning and MS technologies. The results showed that the optimum degradation conditions of PPL for patulin was observed at pH 7.5, 40°C for 48 h. The percentage of degradation could reach above 90%. The structure of degradable product of patulin was inferred by the molecular weight 159.0594, named C_7_H_11_O_4_^+^. It indicated that PPL was effective for the degradation of patulin in fruits and vegetables juice.

## Introduction

**FIGURE 1:**
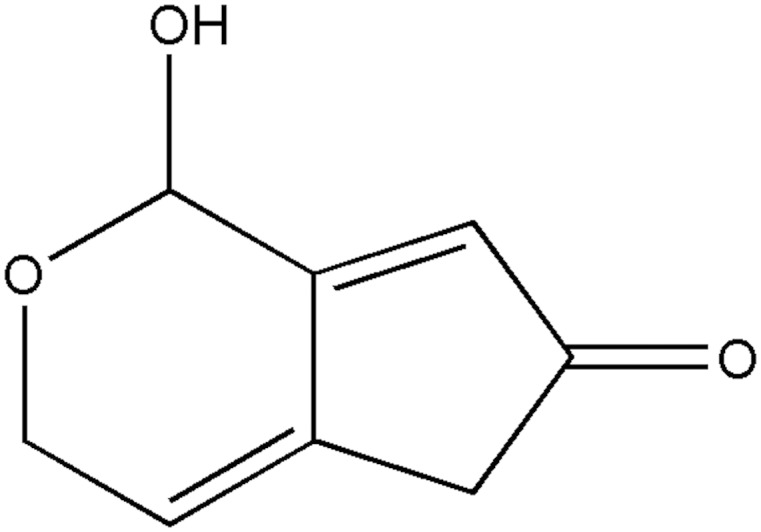
Molecular structure of patulin.

Patulin (4-hydroxy-4H-furo [3, 2c] pyran-2 [6H]-one), a mycotoxin contamination, is synthesized by various fungi, particularly *Penicillium*, *Aspergillus*, and *Byssochlamys* species (Moake et al., 2005; Castoria et al., 2011; Zhu et al., 2015) (**Figure 1**). These fungi are important post-harvest pathogens of apples, pears, peaches, apricots as well as some vegetables (e.g., tomatoes) and caused the accumulation of patulin in infected products (Yuan et al., 2010; Castoria et al., 2011). Patulin poses a health risk to humans and livestock following acute and chronic effects, even at relatively low concentration (Moake et al., 2005; Zhu et al., 2015; Peng et al., 2016). Due to its toxicity, many countries and organizations, including China and WHO, have established the provisional maximum permitted level of patulin contamination for fruit- and vegetable-derived products (Food Agricultural Organization/World Health Organization [FAO/WHO], 1995; Castoria et al., 2005; Yuan et al., 2010). Therefore, it’s necessary to remove patulin from foodstuffs.

The commonly used strategies for patulin removal include filtering and adsorption, electromagnetic irradiation, chemical addition, and biological degradation (Moake et al., 2005; Li et al., 2015). However, some problems still exist in the use of available physical and chemical methods for patulin detoxification, such as safety issues, possible losses in the nutritional quality, environmental damages, limited efficacy, and high cost (Kabak et al., 2006; Guo et al., 2013; Dong et al., 2015). So, the use of biological agents as an alternative strategy is considered as a powerful potential method (Dong et al., 2015). Besides, patulin was nearly degraded completely during the yeast *Saccharomyces cerevisiae* fermentation, this method was more useful than the other decontamination ones (Moake et al., 2005). It has been reported that the biocontrol Yeast *Rhodosporidium kratochvilovae* strain LS11 can reduce patulin contamination in the stored fruit (Castoria et al., 2011) and the complete degradation of patulin was observed within 48 and 72 h in the presence of 15 μg/mL patulin (Reddy et al., 2011). A later study showed that a strain of marine yeast renamed *K. ohmeri* HYJM34 was screened, which has high patulin degradation ability, and the biodegradation of patulin by *K. ohmeri* might be an enzymatically driven process (Dong et al., 2015). However, biological control with yeast is limited to product that it could be fermented in the process (Moake et al., 2005).

The structure of patulin reveals the presence of a lactone bond. Therefore, reducing enzymes such as those involved in yeast fermentation, as well as lactone degrading enzymes such as lactamase, may well be able to degrade patulin alone (Moake et al., 2005). This paper was focus on investigating the effect of enzymes for patulin degradation.

Lipases (triacylglycerol ester hydrolases; E.C. 3.1.1.3) are found in microorganisms, plants and animal tissues. Among them, porcine pancreatic lipase (PPL) is one of the most widely used lipases in catalyzing a variety of reactions, such as esterification, interesterification and hydrolysis, which is cheaper than other commercial microbial and animal lipases (Kartal et al., 2009; Li et al., 2009; Mendes et al., 2012). The PPL investigated is composed of a single chain of 449 kinds of amino acids and 7 kinds of disulfide bonds (Giessauf and Gamse, 2000; Mendes et al., 2012). PPL had already been used as a biocatalyst for enantioselective esterification of glycidol (Martins et al., 1994) and enzymatic hydrolysis of triolein as well as its partial glycerides (Glowacz et al., 1996). In this study, PPL was chosen as a catalyzer that could be possibly used for patulin degradation.

So far, there are few studies about the direct enzymatic degradation of patulin. The aim of this work was to study the degradation of patulin using PPL, which can provide a kind of material to degrade patulin in fruits and vegetables product. And the purpose of this study reported here were to investigate the degradation rate of PPL for patulin at various conditions and characterize the action mechanism by full wavelength scanning and mass spectrometry analysis.

## Materials and Methods

### Materials

The PPL (type II, E.C. 3.1.1.3, with a specific activity of 100–400 olive oil units per milligram of protein) was supplied by Sigma-Aldrich, Co., Ltd. (St. Louis, MO, United States); acetic acid was of analytical purity and used as received without further purification. Acetonitrile and chloroform were of high performance liquid chromatography grade. Patulin was obtained from Sigma-Aldrich, Co., Ltd. Ultrapure water was used throughout all of the experiments.

### Preparation of Patulin Solution

#### Working Solution A

Solid patulin was dissolved into 50 mL of chloroform to obtain 100 mg/L standard patulin solution, and stored at –18°C. The patulin standard solution could evaporated to dry, then dissolved in deionized water (adjusted to pH 4.0 with acetic acid) with the final concentration of 5 mg/L (Zhang et al., 2016). The working solution A was obtained.

#### Working Solution B

The *Penicillium expansum* strain M1 was obtained by our laboratory. Strains M1 was cultivated at 28°C for 14 days in PDA medium. The patulin extraction process was prepared according to the methodology described by MacDonald et al. (2000) with some modifications: the mixture of fungus and culture medium was separated, followed by extracting three times with ethyl acetate, cleaned up by extraction with 10 mL of a 1.5% (w/v) sodium bicarbonate aqueous solution. The ethyl acetate extract was passed over a shaker-incubator with 180 r/min, 25°C for 1 h and evaporated to dryness. Then, patulin was dissolved into 1 mL deionized water, adjusted to pH 4.0 with acetic acid. Thus, the working solution B was obtained.

### Patulin Degradation by PPL

The degradation experiments of patulin in aqueous solution were carried out in 50 mL Erlenmeyer flasks. The powdered PPL was added to 5 mL working solution B constantly. The control was prepared without addition of PPL (Guo et al., 2013). They were placed on a shaker-incubator with 180 r/min, 30°C. The concentration of patulin in aqueous solution after the degradation could be measured by HPLC. Then, 0.45 μm microPES (Shimadzu, Japan) was used for purification before detection (Peng et al., 2016). The samples were detected by HPLC with UV detection (Li et al., 2007).

The effect of lipase amounts on degradation rate was investigated in the range of 0.3–2.4 mg. The effect of pH was investigated at the pH range from 3.5 to 8.5. The pH value was adjusted to the desired 1 mol/L phosphate buffer solution. The effect of temperature on degradation rate was investigated ranging from 20 to 60°C. The effect of contact time was conducted at nine different levels every 6 h for 54 h. The effect of initial patulin concentration was conducted in the range of 5–30 mg/L.

The degradation rate of PPL for patulin was calculated using Eq. (1):

(1) $$\omega \,\;=\;\;\frac{C_{0}\,\;-\;\;C_{e}}{C_{0}}\,\;\;\;\;\;\;\;\;\;\;\;\;\;\;\;\;\;\;(1)$$

where, *ω* (%) is the degradation rate of PPL for patulin; *C_0_* and *C_e_* (mg/L) are the initial and equilibrium concentrations of patulin in the solutions, respectively.

The degradation capacity of PPL for patulin was calculated using Eq. (2).

(2) $$q_{e}\,\;=\;\;\frac{(C_{0}\,\;-\;\;C_{e})\;\;\times \;\;V}{m}\,\;\;\;\;\;\;\;\;\;\;\;\;\;\;\;\;\;\;(2)$$

where, *q_e_* (mg/mg) is the degradation capacity of PPL for patulin; *C_0_* and *C_e_* (mg/L) are the initial and equilibrium concentrations of patulin in the solutions, respectively. *V* (mL) is the volume of patulin aqueous solutions and *m* (mg) is the mass of dry PPL.

### Ultrafiltration and Determination

The Vivaspin centrifugal concentrators with a molecular weight cut off of 3000 were obtained from Millipore (Bedford, MA, United States). The samples were transferred to Vivaspin centrifugal filters spun at 4000 × *g* in swing bucket rotor at 25°C for 10 min to deplete the high molecular weight proteins. Finally, 1 mL of patulin degradation was collected (Zheng et al., 2006).

Then, a 1260 HPLC system (Agilent, United States) equipped with UV detector was used to detect the concentration of patulin. The analytical column was Agilent ZORBAX SB-C18, 5 μm × 4.6 mm × 250 mm; no guard column was used. The mobile phase, eluting at a flow rate of 1 mL/min, consisted of an isocratic mixture of acetonitrile/water (1:9, v:v). The chromatograms for calculations were extracted at 276 nm. The HPLC column was conditioned before analysis by running a background without injection. For regular analysis, 20 μL of sample or standard solution was injected. In addition to samples and calibration standards, control samples were analyzed for each matrix. The requirements for recovery of these samples were set to 60–115%. The limits of detection and quantification were 10.78 and 32.67 μg/L, respectively (Li et al., 2015).

### Identification of the Degradation Products

The powdered PPL was added to 5 mL working solution A constantly.

The optical spectra of samples were recorded by using a Unico UV2102-PC UV-Visible spectrophotometer (Shanghai, China) (Zhu et al., 2016). The samples at 24 h were transferred to Vivaspin centrifugal filters spun at 4000 × *g* for 10 min to deplete the PPL. Finally, 1 mL of patulin degradation product was collected. The preparation of patulin solutions was diluted by ultrapure water. And the UV-vis spectra were recorded from 190 to 700 nm.

Accurate-Mass Q-TOF LC/MS (Agilent, United States) was used. The molecular weight of patulin degradation products was identified by was determined by ESI-MS. The mobile phase eluting at a flow rate of 0.4 mL/min, consisted of an isocratic mixture of methanol/water (1:9, v:v). The sample injection volume was 20 μL. ESI-MS experiments were performed on positive ionization mode. The MS operation parameters were set as followed: capillary voltage 4000 V, drying gas flow 10 L/min, drying gas temperature 350°C, vaporizer temperature 450°C, and nebulizer pressure 40 psi. The optimal fragmentor voltage was 50 V, with a mass range of m/z 20–500 for MS/MS scan modes containing product and precursor ion scans. The Agilent Mass Hunter software package (version 6.1) was used for data acquisition and analysis (Agilent, United States).

### Statistical Analyses

All of the experiments were carried out in triplicate, and the results were expressed as means ± standard deviation. The data was analyzed by one-way analysis of variance (ANOVA) using SPSS (version 19.0, SPSS, Inc.), and Duncan’s multiple comparisons were adopted to assess the statistical significance (*P* < 0.05).

## Results and Discussion

### Effect of PPL Amount on Degradation Rate and Degradation Capacity

**FIGURE 2:**
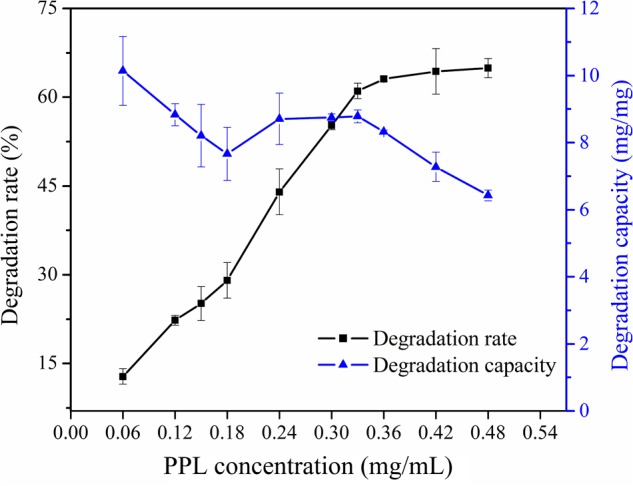
Effect of enzyme concentration on degradation rate and degradation capacity of PPL for patulin.

The dosage of PPL added into 5 mL patulin solution varied between 0.3 and 2.4 mg. Experiments were performed at 30°C for 30 h. As can be seen from **Figure 2**, the degradation rate of patulin increased obviously with the increasing of PPL concentration in solution and approached equilibrium at 0.36 mg/mL. It is more likely to predict that PPL catalyzed the degradation of patulin, while the substrate-binding sites maybe have reached to the saturation point as the concentration of PPL was above 0.36 mg/mL. However, the degradation capacity of PPL was decreased drastically between 0.06 and 0.18 mg/mL, later, it kept invariability. This is probably because that the velocity of PPL promoting reaction was related with the concentration of patulin. Thus, the optimal addition of PPL was 1.8 mg/5 mL.

### Effect of pH on Degradation Rate and Degradation Capacity

**FIGURE 3:**
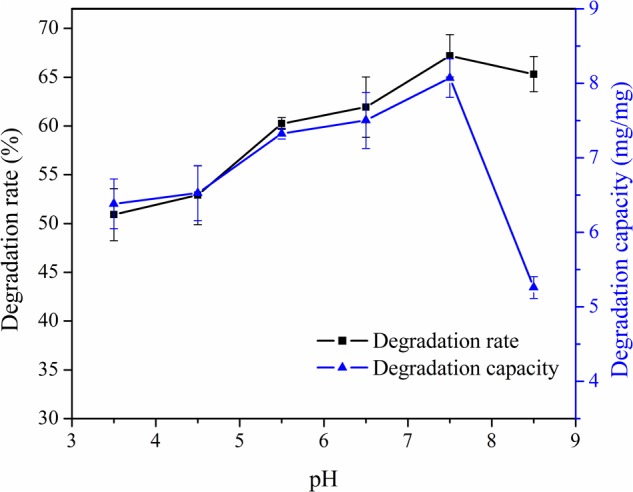
Effect of pH on degradation rate and degradation capacity of PPL for patulin.

Experiments were performed at the controlled pH (3.5–8.5) and 30°C by shaking 1.8 mg of PPL with 5 mL of patulin solutions for 30 h with 180 r/min. Results were shown in **Figure 3**. It indicated that the degradation rate was the highest at pH 7.5. The degradation rate changed insignificantly in the range of 3.5–5.5. This may be explained by the stability of patulin in acidic condition, and meanwhile, the activity of PPL was inhibited. As pH may not only affect the shape of an enzyme, but also it may change the shape or charge properties of the substrate. The data also demonstrated that the degradation rate increased between pH 5.5 and 7.5, later, it changed slightly at high pH. In general, the effect of pH probably results from the activity of enzyme. Therefore, the degradation capacity and degradation rate had the same change trend at early stage, which increased from pH 3.5 to 7.5. However, the degradation capacity declined quickly at pH 8.5. This was because that the activity of PPL was still high, but the patulin content of controlled group was declined significantly for the instability of patulin. Therefore, pH 7.5 was selected as the optimal pH in the following experiments.

### Effect of Temperature on Degradation Rate and Degradation Capacity

**FIGURE 4:**
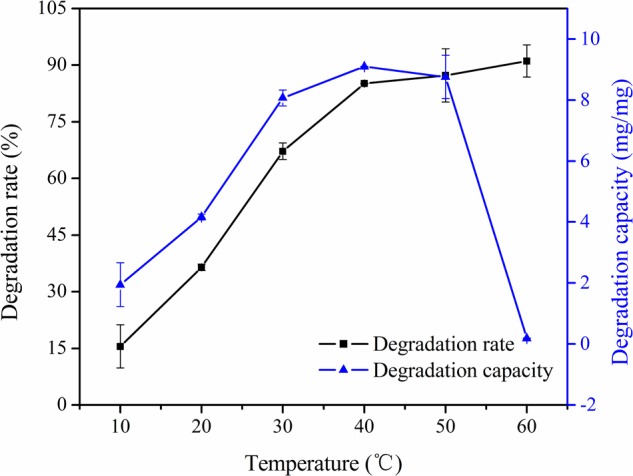
Effect of temperature on degradation rate and degradation capacity of PPL for patulin.

The effect of temperature on degradation rate of PPL for patulin was studied at pH 7.5 and the results were shown in **Figure 4**. The degradation rate increased greatly with an increasing of temperature from 10 to 40°C and then onwards changes slightly. A possible explanation for the results was that the active site of an enzyme was the region that binds the substrates (Berg et al., 2002). The reaction rate would increase with the rising of temperature because the substrates would collide more frequently with PPL active site. And the heat of molecules in the system would increase. Thus, the degradation capacity increased as the temperature raised from 10 to 40°C. The increase of degradation capacity of PPL for patulin with increasing of temperature indicated that the nature of PPL hydrolysis process for patulin was endothermic (Kannamba et al., 2010). Besides, the reaction capacity then abruptly declined with further increase of temperature. This is not only because PPL activity was low, but the patulin content of controlled group was also declined obviously at 60°C. Hence, the optimal temperature was set at 40°C for further studies.

### Effect of Initial Patulin Concentration on Degradation Rate and Degradation Capacity

**FIGURE 5:**
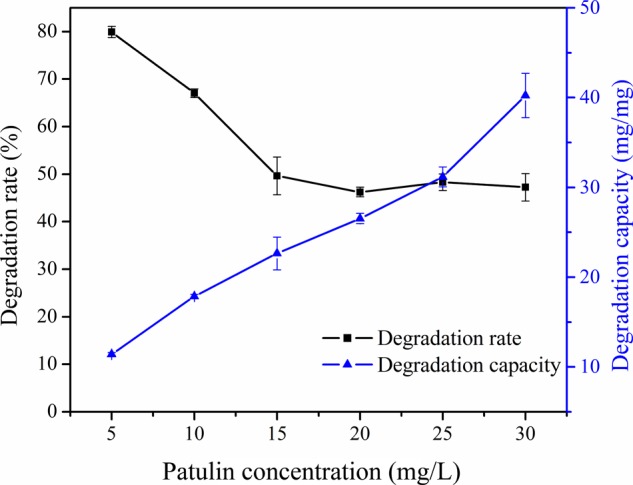
Effect of initial concentration on degradation rate and degradation capacity of PPL for patulin.

Effect of initial concentration of patulin was investigated. Taken into consideration need of practical application, experiments were conducted containing 5–30 mg/L patulin at 40°C for 30 h with 180 r/min (**Figure 5**). The results showed that the degradation rate declined evidently first and then remained steady in varying initial concentration from 15 to 30 mg/L. It was indicated that the degradation effect of patulin was favorable at low substrate concentration. However, the results showed that the degradation capacity increased with the increasing of initial patulin concentration. A possible explanation for this was that PPL was unsaturated with substrate. It showed that the degradation capacity was going to proportional to the concentration of substrate, according to the characteristic of enzymatic reaction (Berg et al., 2002).

### Effect of Contact Time on Patulin Degradation Rate and Degradation Capacity

**FIGURE 6:**
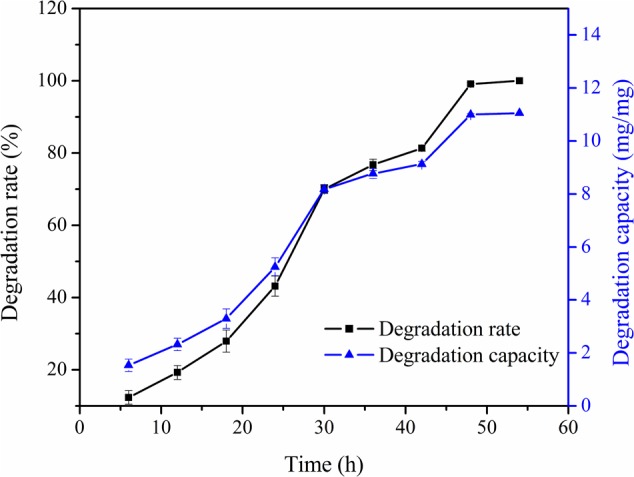
Effect of contact time on degradation rate and degradation capacity of PPL for patulin.

Degradation experiments with PPL were conducted at different time and the results were presented in **Figure 6**. The degradation rate increased with increasing of contact time and reached the maximum value at 48 h. The degradation capacity was following the same trend. The results showed that the rate of degradation increase rapidly with contact time up to 30 h and then onwards was slow considerably. This was because that the active sites of PPL were more and concentration of patulin was higher during the initial stage of degradation (Kannamba et al., 2010; Peng et al., 2016). Similarly, then declining the active sites of PPL limited the reaction rate to the saturation condition.

### Full Wavelength Scanning

**FIGURE 7:**
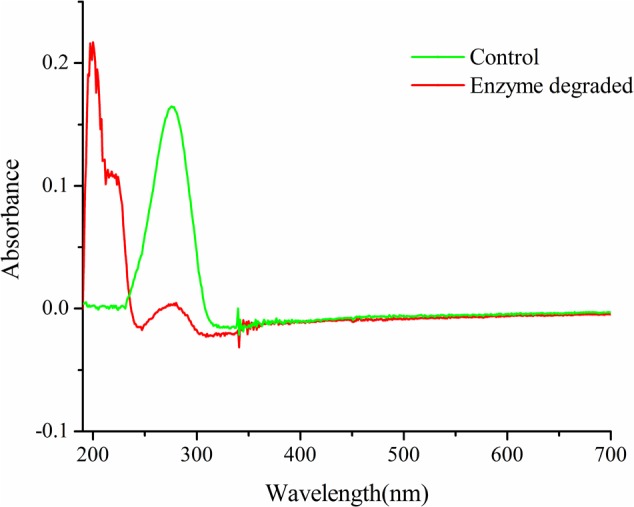
The spectrum of the response result reactions with patulin and PPL.

**Figure 7** showed the degradation spectrum of patulin at 24 h. The relationship between wavelength of the maximum absorption and structure was also explained (Ibarz et al., 2014). The results showed that the maximum absorption peaks of degradation product shifted to shorter wavelengths. And there was typical absorption of conjugated structures by UV scanning.

### MS Scan of Degradation Products

**FIGURE 8:**
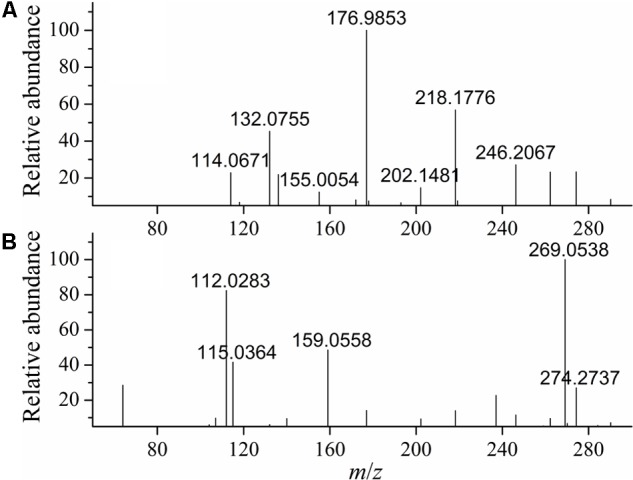
MS/MS before **(A)** and after **(B)** PPL degradation.

**FIGURE 9:**
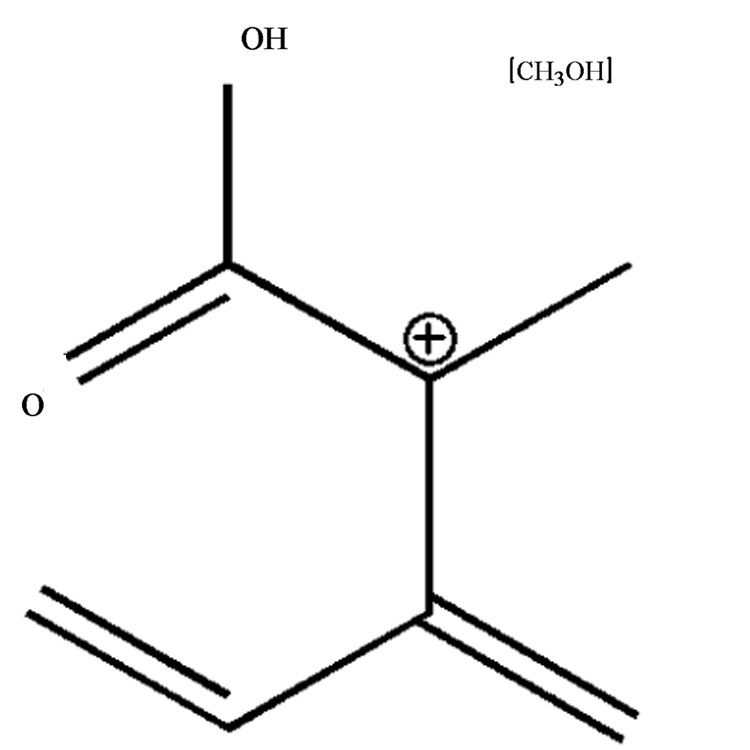
The structure of degraded product of patulin.

MS analyses of patulin before and after PPL degradation were shown in **Figure 8**. As was shown in **Figure 8A**, the standard aqueous solution of patulin with Na^+^ adducts C_7_H_6_NaO_4_ (M+Na)^+^ calculated: 177.0158, the ESI-MS found: 176.9852. And patulin was identified at m/z = 155.0054 for protonated cation [M+H]^+^. MS of patulin after degradation (**Figure 8B**) showed that the patulin in PPL treated samples was very less than untreated ones. The molecular weight of product might be 159.0558, according with the molecular weight 159.0594 for C_7_H_11_O_4_^+^ (**Figure 9**). It indicated that patulin was reacted with ring opening reaction with PPL. The fragment at m/z 159 can alternatively undergo successive losses of carbon dioxide (Malysheva et al., 2012). The speculation corresponds to the previous study by UV scanning. It was suggested that patulin was possibly metabolized to degradation product, which was chemically different from patulin.

## Conclusion

In this study, PPL has been successfully used to degrade patulin in aqueous solution. It was conjectured that the process of patulin degradation was enzymatic reaction. Batch studies showed that the degradation percentage of patulin was strongly dependent on reactive conditions such as pH, temperature, initial patulin concentration, and contact time. The complete degradation of patulin occurred at pH 7.0, 40°C for 48 h, the degradation capacity of PPL for patulin is 10.99 mg/mg PPL. The mechanism of degradation was discussed by using full spectrum scanning and MS analysis. Generally, PPL exhibited good degradation ability and it might have practical application for degradation of patulin in apple juice.

## Author Contributions

BL did the experiments and organized the manuscript. XP did the experiments. XM guided the analysis of the catabolite structure.

## Conflict of Interest Statement

The authors declare that the research was conducted in the absence of any commercial or financial relationships that could be construed as a potential conflict of interest.
